# 以ETV6/RUNX1探针为例探讨FISH性能验证方法

**DOI:** 10.3760/cma.j.cn121090-20230721-00015

**Published:** 2024-01

**Authors:** 静 肖, 迎春 郑, 佳炜 赵, 成华 崔, 慧君 王, 琦 孙, 娇 马, 跃申 马, 振 宋, 志坚 肖, 承文 李

**Affiliations:** 1 中国医学科学院血液病医院（中国医学科学院血液学研究所），血液与健康全国重点实验室，国家血液系统疾病临床医学研究中心，细胞生态海河实验室，天津 300020 Institute of Hematology & Blood Diseases Hospital, Chinese Academy of Medical Sciences & Peking Union Medical College, State Key Laboratory of Experimental Hematology, National Clinical Research Center for Blood Diseases, Haihe Laboratory of Cell Ecosystem, Tianjin 300020, China; 2 天津医学健康研究院，天津 301600 Tianjin Institutes of Health Science, Tianjin 301600, China

**Keywords:** 荧光原位杂交, 灵敏度, 特异性, 诊断符合率, 实验室自建方法, fluorescence in situ hybridization, Sensitivity, Specificity, The diagnostic coincidence rate, Laboratory developed test

## Abstract

**目的:**

探讨FISH探针应用于临床检测前的规范化性能验证。

**方法:**

选取20名正常供者检测后剩余的骨髓样本进行间期细胞和中期分裂象FISH杂交，分析ETV6/RUNX1探针的灵敏度和特异性。采用反β函数建立该探针的阈值体系，并制定判读标准。最后以PCR检测为对照组，对286例中国医学科学院血液病医院患者骨髓样本进行平行对照研究，分析ETV6/RUNX1探针FISH检测的临床敏感性、特异性、诊断符合率，并采用Kappa检验分析两种方法诊断一致性。

**结果:**

ETV6/RUNX1探针灵敏度为98.47％，特异性为100％。计数50、100和200个细胞时经典阳性信号阈值分别为5.81％、2.95％和1.49％，非经典阳性信号阈值分别为13.98％、9.75％和6.26％。FISH检测临床敏感性为96.1％，特异性为99.6％，诊断符合率为99.00％，诊断一致性检验Kappa值为0.964，*P*值<0.001。

**结论:**

不具备国家医疗器械注册证的商品化FISH探针，可通过实验室自建方法验证标准进行探针的规范化性能验证和方法学性能确认，以保证为临床诊疗提供可靠、精准的参考依据。

## Abstract (en)

**Objective:**

To explore the standardized performance of a FISH probe before clinical detection.

**Methods:**

The probe sensitivity and specificity of ETV6/RUNX1 were analyzed via interphase and metaphase FISH in 20 discarded healthy bone marrow samples. The threshold system of the probe was established using an inverse beta distribution, and an interpretation standard was established. Finally, a parallel-controlled polymerase chain reaction detection study was conducted on 286 bone marrow samples from patients at our hospital. The clinical sensitivity, specificity, and diagnostic coincidence rate of ETV6/RUNX1 FISH detection were analyzed, and the diagnostic consistency of the two methods was analyzed by the kappa test.

**Results:**

The probe sensitivity and specificity of the ETV6/RUNX1 probe were 98.47% and 100%, respectively. When 50, 100, and 200 cells were counted, the typical positive signal pattern cutoffs were 5.81%, 2.95%, and 1.49%, respectively, and the atypical positive signal pattern cutoffs were 13.98%, 9.75%, and 6.26%, respectively. The clinical sensitivity of FISH was 96.1%, clinical specificity was 99.6%, diagnostic coincidence rate was 99.00%, diagnostic consistency test kappa value was 0.964, and P value was <0.001.

**Conclusion:**

For FISH probes without a national medical device registration certificate, standardized performance verification and methodology performance verification can be performed using laboratory developed test verification standards to ensure a reliable and accurate reference basis for clinical diagnosis and treatment.

FISH技术是分子细胞遗传学的主要临床检测手段，可直接观测单个细胞核内染色体的异常情况，并如实反映所检测克隆的类型、大小；能快速、灵敏地检测染色体的数目和结构异常，可弥补传统细胞遗传学的不足[1]–[2]。该技术已成为常规细胞遗传学检测的有益补充手段之一[3]。

近年随着临床检测需求量不断增大，FISH探针品牌、种类越来越多，但具备国家医疗器械注册证的产品相对较少，无法满足日益增长的临床需求。本研究以ETV6/RUNX1探针为研究对象，参考实验室自建方法（laboratory developed tests, LDT）的验证流程，探讨临床实验室采用此类不具备国家医疗器械注册证的商品化FISH探针时，如何规范化进行探针性能验证和临床诊断性能确认[4]–[6]。

## 材料与方法

一、探针杂交性能验证

FISH探针为雅培公司LSI ETV6（TEL）/RUNX1（AML1）ES双色单融合探针，ETV6（12p13）基因标记为绿色，RUNX1（21q22）基因标记为红色。样本来源为20名中国医学科学院血液病医院（中国医学科学院血液学研究所）2020年11月1日至2020年12月31日待移植正常供者完成检测后剩余的骨髓样本（CIFMS2022011-EC-2）。

1. 探针灵敏度（probe sensitivity）：将20名供者的骨髓样本，制备细胞悬液滴片，进行ETV6/RUNX1探针FISH杂交，每名分析200个间期细胞，计数呈现红色和绿色荧光信号的间期细胞与计数细胞总数（200个）的比例。随机从20份样本中选取10份样本，每份分别选取5个中期分裂象，合计50个中期分裂象进行杂交，计数12号和21号染色体分别显示绿色和红色荧光信号的中期分裂象比例。从而分别得到在间期细胞和中期分裂象中ETV6/RUNX1探针的灵敏度。

2. 探针特异性（probe specificity）——中期FISH：分析计数如上杂交的50个中期分裂象，将绿色荧光信号在12p13位点的个数和红色荧光信号在21q22位点的个数之和除以所有染色体上看到的红色和绿色荧光信号总数，从而得到ETV6/RUNX1探针特异性。

二、阈值计算

以上完成杂交的20份正常骨髓样本，每张玻片由2名检测人员分别计数25、50和100个细胞，分别记录下所有信号的类型和个数，采用逆β函数计算出计数50、100和200个细胞时ETV6/RUNX1探针的阈值，具体公式为：BETA.INV（置信区间95％，观察到的最大的假阳性细胞数+1，总计数细胞）。根据探针设计，荧光信号2R2G为阴性信号，1F2R1G（F-融合信号，R-红色荧光信号，G-绿色荧光信号）为经典阳性信号，除经典阳性信号以外，凡具备融合信号的信号模式（如1F1R1G、1F1～2R、1F1～2G、2F1R1G等）为非经典阳性信号。

三、临床诊断性能确认

样本来源为2021年1月1日至2022年3月31日就诊我院，临床表现、细胞形态学以及免疫分型结果疑诊急性淋巴细胞白血病患者的初诊骨髓样本。

1. 试验设计：采用平行对照的试验设计，ETV6/RUNX1探针FISH检测为试验组，ETV6::RUNX1融合基因PCR方法检测为“金标准”对照组[7]。患者的同一份生物样本分别接受试验组和对照组的检测，将二者结果进行对比分析。由于二者的操作完全不一样，也无法实现盲法设计，故采用开放式试验设计。

参考2021年9月颁布的《体外诊断试剂临床试验技术指导原则》，根据探针性能验证所得探针灵敏度和特异性，采用敏感性置信区间方法计算样本量[5]，设置置信水平95％，区间宽度5％，得到阳性样本数量不低于49例。同一骨髓样本抽取后同时进行 ETV6/RUNX1探针FISH检测、ETV6::RUNX1融合基因PCR检测，并参考同期流式免疫分型检测结果，要求异常细胞需大于0。共计286例，男163例（56.99％），女123例（43.01％），纳入受试者中位年龄为7（0～55）岁。

2. 数据统计：按照定性诊断试剂的相关统计要求，本研究统计分析检验均采用双侧假设检验。本次研究为比较类型，包括统计描述和诊断一致性检验。统计描述为计算并汇报试验试剂相对于对照方法的临床敏感性（clinical sensitivity）、特异性（clinical specificity）、诊断符合率、阳性预测值和阴性预测值及其95％*CI*。诊断一致性检验使用二分类资料一致性分析的Kappa检验。统计性能分析参数定义及公式见诊断四格表[6]（表1）。

**表1:** 诊断四格表

试验组（FISH）	对照检测方法（PCR法）		总数
	阳性	阴性	
阳性	TP	FP	TP+FP
阴性	FN	TN	FN+TN
总数	TP+FN	FP+TN	N

注 TP：真阳性数；FP：假阳性数；FN：假阴性数；TN真阴性数；统计性能分析参数计算公式：（1）$敏感性（\text{SE}）＝\frac{TP}{TP+FN}\times 100\text{\%}$；（2）$特异性（\text{SP}）＝\frac{TN}{FP+TN}\times 100\text{\%}$；（3）$诊断符合率＝\frac{TP+FP}{TP+FP+FN+TN}\times 100\text{\%}$；（4）$阳性预测值（\text{PPV}）=\frac{TP}{TP+FP}\times 100\text{\%}$；（5）$阴性预测值（\text{NPV}）=\frac{TN}{FN+TN}\times 100\text{\%}$

3. 判读流程：每份样本分不同区域计数并分析200个细胞，分别计数各荧光信号的类型和数量。根据计数及分析的细胞数量、信号类型，以及相应的阈值判断样本的阴阳性，具体判断流程见图1。不同类型的信号按照各自的阈值判断。当细胞计数总数不足200个，则应选择计数分析50或100个细胞时对应的阈值。

**图1:**
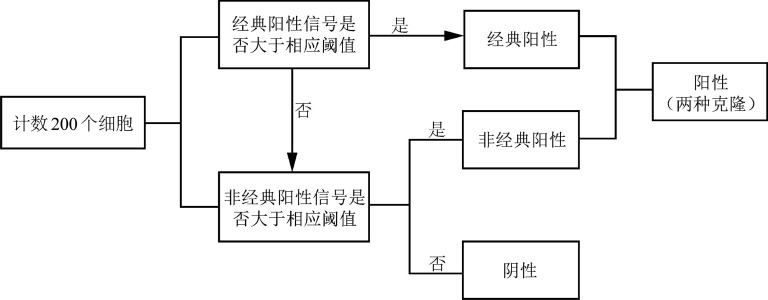
ETV6/RUNX1探针判读流程示意图（计数200个细胞）

四、统计学处理：

统计分析所用软件IBM SPSS（21.0及以上版本），MedCalc（16.2及以上版本）和EpiData（3.02版本）。描述试验用体外诊断试剂的诊断一致率及其95％*CI*，Kappa检验，*κ*值应大于0.75，且假设检验结果应*P*<0.05。一致性检验为两分类的Kappa检验，检验假设如下：*H*0 *κ*＝0；*H*1 *κ≠*0；在α＝0.05，1−*β*＝0.8水平，当*P*<0.05，则认为组间一致性具有统计学意义；当*P*≥0.05，认为组间一致性不具有统计学意义。本次试验仅为一次性诊断检测，不涉及多次诊断或治疗，故此不进行基线特征分析，且无依从性和合并用药分析。

## 结果

一、探针的性能研究结果

1. 探针灵敏度：20份正常骨髓细胞样本，每个样本计数200个细胞，共计数4 000个细胞，其中3 939个细胞显示红绿荧光信号，ETV6/RUNX1探针对间期骨髓细胞在我院进行FISH检测所得探针灵敏度为98.47％。10个正常样本合计分析50个中期分裂象，50个分裂象的12和21号染色体上都显示红绿色荧光信号，ETV6/RUNX1探针对中期分裂象进行检测所得探针灵敏度为100％。

2. 探针特异性——中期FISH：分析50个中期分裂象，位于12p13上的ETV6位点显示绿色荧光信号，位于21q22上的RUNX1位点显示红色荧光信号，无其他染色体位点之间出现交叉信号，该探针在我院进行FISH检测的特异性为100％。

本研究ETV6/RUNX1探针的灵敏度为98.47％，特异性为100％。通常FISH探针灵敏度≥95％，特异性≥98％，即可用于临床检测。

二、阈值

20份正常骨髓细胞样本，分别计数50、100和200个细胞时，经典阳性信号可见最大假阳性细胞数为0，采用逆β函数计算，所得经典阳性信号阈值分别为5.81％、2.95％和1.49％；非经典阳性信号可见最大假阳性细胞数分别为3、5和7，计数所得非经典阳性信号阈值分别为13.98％、9.75％和6.26％。详见表2。

**表2:** ETV6/ RUNX1探针阈值

骨髓样本	计数不同细胞数目经典阳性信号			计数不同细胞数目非经典阳性信号		
	50	100	200	50	100	200
1	0	0	0	0	1	3
2	0	0	0	1	1	3
3	0	0	0	1	1	4
4	0	0	0	2	3	4
5	0	0	0	1	1	3
6	0	0	0	1	2	4
7	0	0	0	0	2	4
8	0	0	0	3	5	7
9	0	0	0	0	0	1
10	0	0	0	3	5	7
11	0	0	0	0	0	1
12	0	0	0	1	3	5
13	0	0	0	2	4	6
14	0	0	0	1	2	3
15	0	0	0	1	2	4
16	0	0	0	1	2	2
17	0	0	0	0	2	5
18	0	0	0	0	1	3
19	0	0	0	2	4	4
20	0	0	0	2	4	5
阈值（%）	5.81	2.95	1.49	13.98	9.75	6.26

注 经典阳性信号：1F2R1G；非经典阳性信号：计数过程中可见1F1R1G、1F1R、1F1G、2F1R1G

三、临床诊断性能确认结果

根据PCR对照组检测结果，可得阳性样本数为51例（17.8％，51/286），阴性样本数为235例（82.2％，235/286）。FISH试验组检测结果，阳性50例，其中49例（98％）与对照组结果一致，阴性236例，其中234例（99.2％）与对照组结果一致，详见表3。

**表3:** FISH检测结果与PCR检测结果描述［例（％）］

FISH检测结果	PCR检测结果		合计
	阳性	阴性	
阳性	49（98.0）	1（2.0）	50（100）
阴性	2（0.8）	234（99.2）	236（100）

合计	51（17.8）	235（82.2）	286（100）

根据诊断四格表可知真阳性数（TP）为49例，真阴性数（TN）为234例，假阳性数（FP）为1例，假阴性数（FN）为2例，根据统计性能分析参数定义及计算公式可得试验组与对照组的检测符合率为99％（95％ *CI* 97.0％～99.8％），该探针FISH检测的临床敏感性为96.1％（95％ *CI* 86.5％～99.5％），临床特异性为99.6％（95％ *CI* 97.7％～100％），阳性预测值为98.0％（95％ *CI* 89.4％～99.9％），阴性预测值为99.2％（95％ *CI* 97.0％～99.9％）。

283例样本（98.95％）试验组与对照组诊断结果一致，有3例（1.05％）诊断结果不一致，诊断一致率为99％（95％ *CI* 97％～99.8％）。诊断一致性检验采用Kappa检验分析，*κ*值为0.964（95％ *CI* 1～0.923）（κ≥0.75），同时*P*<0.001，组间一致性有统计学意义。研究结果显示FISH检测结果和PCR检测结果之间存在一致性，证明采用该探针进行FISH检测具有较高的诊断价值且检测结果准确可信。

本研究通过对ETV6/RUNX1探针杂交性能、检测分析能力和临床诊断能力三方面的性能评价，探讨了不具备医疗器械注册证的商品化FISH探针应用于临床检测前的规范化性能验证方法，并确认可行后续将不断完善并推广。

## 讨论

LDTs是经国家药监局许可或批准，由医疗机构和（或）第三方实验室自行研制（也可使用购买或自制的试剂或试剂盒），且仅在本机构使用的检测方法。在应用于临床检测前，对相应的性能指标进行验证和确认，如精密度、准确度、线性范围、参考区间、分析灵敏度、分析特异性、临床敏感性和临床特异性等。国内目前尚无FISH检测相关实验室验证指南，参考美国临床检验标准化委员会颁布的《临床实验室荧光原位杂交方法研究指南第二版》，FISH探针用于临床实验室检测前，应从探针杂交性能、检测分析能力以及临床诊断能力三个维度进行评估[8]–[10]。

探针的杂交性能包括探针灵敏度和特异性两个方面。探针灵敏度是测试探针与靶标染色体或互补DNA结合的杂交效率，即具有预期探针信号的可分析间期细胞核或中期分裂象与预期信号的百分比。完成杂交的样本中，存在无荧光信号的间期细胞核或中期分裂象，可能是由于细胞凋亡导致，也可能是探针无效杂交导致，可通过分析有或无荧光信号细胞的比例来评估探针杂交的敏感性。探针特异性则是衡量探针是否只结合其预定目标的能力，是在预期位置发生所有可参与计数信号的百分比。通过中期FISH，分析荧光信号出现在目标染色体位点的百分比，从而评估探针杂交特异性。

在检测分析能力确认方面包括分析灵敏度（analytical sensitivity）和分析特异性（analytical specificity）。分析灵敏度可以指一个测试能够检测到的最小量的测量物，也可以指一个测试能够检测到的最小量的测量物的变化。前一个描述与检测限（LoD）同义，而临床化学中常用的计算LoD的方法不适用于FISH检测，因为FISH检测的对象是细胞内的信号模式，没有所谓的“空白”样本，且细胞悬液不是均匀混合物，目前也没有测量技术能准确量化异常细胞的“浓度”，所以通常难以获得异常细胞的百分率接近LoD的细胞混合物[11]–[12]。FISH检测关注的检测限，是预计在正常对照组中发现的异常FISH信号模式的最高百分率，也就是计算正常细胞中异常信号模式的上限，此上限构成“正常截止值”（cut-off值），即“阈值”。分析特异性是衡量一个测试只检测其目标测量物的能力。FISH检测的测量物是信号模式，FISH探针分析特异性是衡量FISH试验在存在与之相关的异常信号模式时，判断异常信号模式检测呈阳性的百分率。一个FISH检测常常同时分析大量信号模式的存或不存在。所以在建立阈值时须根据探针设计对各种信号模式分别建立阈值。

FISH阈值以往常用的计算方法是*x*±*s*，该公式描述的是均值上下两个方向。FISH分析是定性分析，报告为阳性或阴性，是个二元函数，所以使用二项分布的单侧95％置信区间的上限作为确定FISH的阈值的计数方式更为合适，即反β函数。需要注意的是，FISH探针的阈值是通过计数部分正常人群假阳性信号细胞数计算所得，在阈值附近的计数结果都可能存在假阳或假阴的风险。对于初诊患者来说，大多数FISH结果显示大量的阳性信号细胞，较容易判断阴阳性。然而，接受治疗的患者或复查的患者，FISH检测计数结果可能会产生接近阈值的情况，这是FISH检测的“灰区”，此时我们不仅需要扩大计数细胞数量（也可增加一名计数人员进行计数分析），还需要结合临床其他相关信息，发出准确的报告[13]–[14]。

FISH检测的分析能力不仅与探针性能相关，还与实验室的操作条件和人员密切相关，所以每个实验室都应建立自己探针的阈值体系。FISH探针的阈值应仅适用于本实验室，这也符合LDTs的要求。

最后是进行临床诊断性能确认，即衡量有特定临床症状的患者进行ETV6/RUNX1探针FISH检测后确定为ETV6::RUNX1阳性患者的百分率。其中有2例PCR检测结果为阳性，FISH检测结果为阴性，这2例中1例PCR检测阳性率0.01％，1例流式细胞术检测异常细胞比率为0.16％，考虑是分析灵敏度差异导致对照组与实验组结果不一致。PCR技术是分子水平的检测，FISH技术是在细胞水平进行检测，其分析灵敏度低于PCR技术，且样本中异常细胞数量低于阈值时则超出FISH检测分析灵敏度。另有1例PCR检测结果为阴性，FISH检测结果为阳性（1F2～3R），考虑是引物设计缺陷导致结果差异。FISH技术是检测ETV6和RUNX1基因的DNA结构融合，PCR技术检测的是ETV6::RUNX1融合基因产生的mRNA，此外，FISH探针覆盖DNA片段较大，一般为几百kb，PCR检测往往需要多对引物才能覆盖所有的断裂重排位点，当遇到RNA提取质量不佳或引物未能覆盖到的断裂重排位点的样本时，两种检测方法可存在检测结果不一致[15]–[18]。

FISH检测项目在临床实验室的开展，不仅要按照标准化的步骤进行试验操作，对检测人员的规范化培训也是保证检测结果科学、准确的必要条件。首先在新项目开展前，需要对人员进行探针说明书、操作流程、杂交信号判读进行培训。随后对新探针进行临床应用前验证时，检测人员应充分参与探针性能验证、阈值建立，规范实验室检测人员对该探针的判断标准和判读流程，并按照ISCN指南进行结果描述，建立本实验室统一的报告模板。在临床性能验证过程中以及后期临床检测过程中，检测人员应遵守标准化的操作流程，做好室内、室间人员比对，以及定期进行检测人员培训，从而保证检测质量的延续性。
